# The application of artificial intelligence in spine surgery

**DOI:** 10.3389/fsurg.2022.885599

**Published:** 2022-08-11

**Authors:** Shuai Zhou, Feifei Zhou, Yu Sun, Xin Chen, Yinze Diao, Yanbin Zhao, Haoge Huang, Xiao Fan, Gangqiang Zhang, Xinhang Li

**Affiliations:** ^1^ Department of Orthopaedics, Peking University Third Hospital, Beijing, China; ^2^ Engineering Research Center of Bone and Joint Precision Medicine, Peking University Third Hospital, Beijing, China; ^3^ Beijing Key Laboratory of Spinal Disease Research, Beijing, China

**Keywords:** artificial intelligence, application, spine surgery, machine learing, treatment

## Abstract

Due to its obvious advantages in processing big data and image information, the combination of artificial intelligence and medical care may profoundly change medical practice and promote the gradual transition from traditional clinical care to precision medicine mode. In this artical, we reviewed the relevant literatures and found that artificial intelligence was widely used in spine surgery. The application scenarios included etiology, diagnosis, treatment, postoperative prognosis and decision support systems of spinal diseases. The shift to artificial intelligence model in medicine constantly improved the level of doctors' diagnosis and treatment and the development of orthopedics.

## Introduction

As a new subject, artificial intelligence (AI) mainly studies a new technology for imitating and expanding human intelligence. In the past 10 years, AI has made tremendous progress. Machine learning (ML) is a subset of AI that enables algorithms or classifiers to learn large complex data sets and generate useful predictive outputs. More specifically, common applications of ML include classification, regression and clustering. Another way to describe the different forms of ML is based on the nature of the tasks to be performed which include supervised learning, unsupervised learning and reinforcement learning (1). And supervised learning is the most common type of learning used in medical research. The methods used for supervised learning are briefly described in Table 1.

**Table 1:** The methods used for machine learning.

	Description	Feature
Linear regression	Fitted by means of the least squares method	Simplicity; Incapability of capturing a nonlinear behavior; Underfitting
Logistic regression	Seen as the equivalent of linear regression for classification problems	Multiclass classification problems
Bayes classifier	Based on Bayes’ theorem of conditional probability	Simplicity
Support vector machine	Build the hyperplane, or a number of them, which can divide the space so that the points of the different classes are effectively and optimally partitioned	Multiclass linear classification tasks, including image segmentation; Adapted to nonlinear classification and regression problems
Decision trees	Link the values of the features to the possible outputs, therefore implementing a classification or a regression task, by means of a set of conditions	Easier to understand; Suitable for very large datasets
Artificial neural networks	Resemble how the neurons are connected and interact in the brain	Reduce the risk of overfitting; Achieve a faster and more robust convergence
Convolutional neural networks	Mimic the structure of the animal visual cortex	Image processing; Reduce the risk of overfitting

This study reviewed the scientific literature from 2007 to 2022 with syntax specific for machine learning and spine surgery applications. Articles not available in the full text were excluded, as well as duplicate articles and those that did not utilize a form of AI or ML pertaining to spine surgery. Specific data was extracted from the available literature including algorithm application, algorithms tested, database type and size, algorithm training method, and outcome of interest. A total of 49 studies met inclusion criteria and our interest. Studies were grouped into five general types:etiology, diagnosis, treatment, postoperative prognosis and decision support systems of spinal diseases. Across studies, a wide swath of algorithms were used, which were trained across multiple disparate databases.

**Figure 1:**
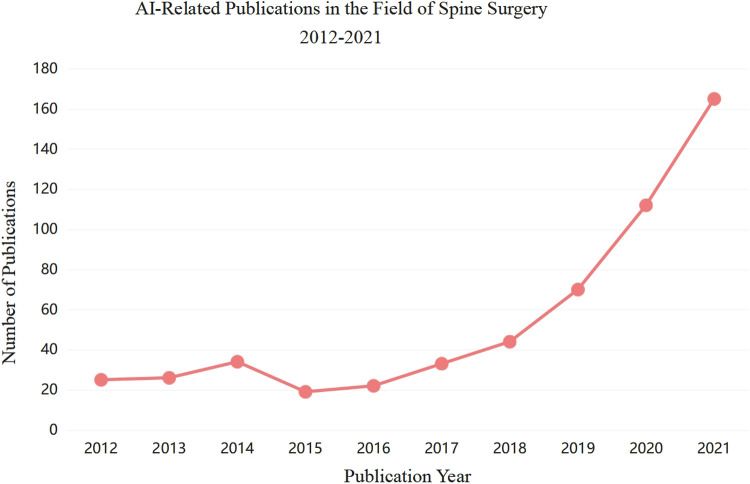
AI-related publications in the field of spine surgery 2012–2021.

As testified by the sharp increase in the number of published papers in recent years, AI and ML are more and more being used in the field of spine surgery (Figure 1). Starting from whether the new technology of artificial intelligence can have an impact on the whole process of traditional spinal disease diagnosis and treatment, this paper intends to review the application of artificial intelligence in spinal surgery from the whole process of etiology, diagnosis, treatment, postoperative prognosis and decision support systems of spinal diseases, make use of clinical transformation platform to break through cutting-edge medical technology, standardize the diagnosis and treatment plan of spinal diseases, strive to obtain more original research results with practical application value or theoretical significance, and make contributions to the protection of people's health and safety.

## Etiology

With changes in social lifestyles, the incidence of spinal diseases is increasing. Medical workers have been committed to the etiology of spinal diseases for a long time, because only when the etiology is determined, can we better prevent the occurrence of diseases. In the field of spine surgery, the etiology is often closely related to the patient's personal, environmental, social and other factors. Therefore, the advantage of machine learning in processing large data can better analyze the etiology. For example, as a prevalent degenerative disease of the cervical spine, the trend of the loss of the physiological cervical spine curve has not been fully defined across gender and age groups. In 2020, Shin (2) used an automated deep learning system (DLS) to conduct a population-based large-scale epidemiological study of cervical curvature. Lateral radiographs of 13,691 patients were analyzed with automatic cervical spine segmentation, From 2006 to 2018, the decline in the lordosis curve was prominent in both men and women under 70 years of age and in age groups, and the decline was more remarkable in women and the younger. This rapid decline for women might be related to the increase in mobile-centric environments over the past decade and the increase in smartphone addiction (3). Further studies are needed to assess the association between neck pain and loss of cervical lordotic curve to meet the need for neck postural correction.

Electronic medical record systems are a key source of medical data, generating large and expanding data sets. Rich data stored in electronic medical record system and computer processing power are in good agreement with the development of artificial intelligent technology, in the future, spine disease etiology analysis will more come from patients' electronic health records database or national health insurance database. Due to the processing ability of artificial intelligence to huge data, the etiology can be better analyzed, so as to achieve the early intervention of pathogenic factors and provide help for follow-up treatment.

## Diagnosis (based on imaging)

Medical imaging plays an important role in the diagnosis and treatment of spinal diseases. Automatic detection, classification and location of disease in medical images are significant tasks to support clinical decision. The increasing burden of spinal disease related to an aging population and the increasing availability of magnetic resonance imaging (MRI) and computed tomography (CT) scans have led to a significant increase in radiological data related to the spine. Artificial intelligence (AI) and machine learning (ML) technologies have also made significant advances in recent years, automated analysis capabilities of machine learning models can quickly generate quantitative parameters from image data, which will reduce radiologists' workload. In fact, for the analysis of spinal imaging data, locating anatomical structures in the imaging data is usually the first step in the progress of fully automated analytical methods for detecting, classifying, or predicting pathological features. For example, Schmidt (4) uses the classification tree method to produce the probability map of the centroid position of each intervertebral disc in MRI images, and then uses the probability map model to infer the most likely position of the intervertebral disc centroid. Compared with manual measurement, the average positioning error is 6.2 mm. Oktay and Akgul (5) trained the model for disc localization using support vector machine (SVM) and achieved an average positioning error of 2.6–3.6 mm based on disc level. At the same time, the author also improved the method to locate the vertebral body, and achieved an average positioning error of less than 4 mm (6). Glocker (7, 8) used the random forest method to establish a model for locating the vertebral body in CT images with an average positioning error of 6–8.5 mm, thus solving the difficult problems of locating the vertebral body in CT images of pathological spinal diseases, including serious scoliosis, sagittal deformity and the presence of internal fixation. Recently, artificial neural network and deep learning have also been used to locate spinal structures. Chen (9) established an artificial neural network model to predict the intervertebral disc centroid, making the average positioning error reduce to 1.6–2 mm, which is a significant improvement over previous models not based on deep learning. In 2021, Suri (10) developed a deep learning system that can automatically and fastly segment vertebral body and discs in MR, CT, and x-ray imaging studies. The model was able to produce median Dice scores >0.95 in all modalities for vertebral bodies and intervertebral discs. Radiomic features calculated from predicted segmentation masks were highly accurate (*r* ≥ 0.96 across all radiomic features when compared to ground truth). Mean time to produce outputs was <1.7 s in all modalities. The model can be immediately used in radiological and clinical imaging studies to assess spinal disease, because it can quickly produce the output of these commonly used modalities. The most advanced technology for locating and mapping spinal structures on imaging are now comparable to those of human experts.

In the future, ML models may combine clinical information with quantitative parameters from patient imaging information, such as patient demographic information and neurological examination. It can provide decision making to clinicians. This decision tool uses machine learning technology to determine which patients will benefit from surgery and help with surgical planning. This has led to a quick increase in research connected with computer-assisted spinal imaging analysis (Table 2).

**Table 2:** AI and ML in the diagnosis of spinal diseases.

Author	Models	Dataset	Type of outcome	Result
Schmidt et al. (4)	Probability map	16 images	Intervertebral disc centroid	Average positioning error 6.2 mm
Oktay et al. (5)	SVM	40 subjects/240 discs	Disc localization	Average positioning error 2.6–3.6 mm
Oktay et al. (6)	SVM	80 subjects/400 lumbar vertebrae	Vertebral body	Average positioning error less than 4 mm
Glocker et al. (7)	Random forest	200 CT scans	Vertebral body	Average positioning error 6–8.5 mm
Glocker et al. (8)	Random forest	424 CT scans	Vertebrae localization	Average positioning error 6–8.5 mm
Chen et al. (9)	ANN	35 patients/245 discs	Intervertebral disc centroid	Average positioning error 1.6–2 mm
Suri et al. (10)	ANN	1,123 MR, 137 CT, 484 x-ray	Vertebral bodies and intervertebral discs	Median Dice scores >0.95
Carson et al. (11)	CNN	50 subjects	Detect anatomic structures	Mean Dice coefficient score for each tissue type was >80%
Galbusera et al. (12)	CNN	493 patients	Predict spine shape	2.7°–11.5°
Korez et al. (13)	CNN	55 subjects/97 images	Parameters of the sagittal spinopelvic balance	No statistically significant differences
Yeh et al. (14)	CPN	2,210 images	Anatomic landmarks	Matches the reliability of doctors for 15/18
Wu et al. (15)	MVC-Net	154 patients/526 images	Adolescent Idiopathic Scoliosis (AIS)	4.04° CMAE in AP Cobb angle and 4.07° CMAE in LAT Cobb angle
Tomita et al. (16)	CNN	1,432 CT scans	Extract radiological features	Accuracy of 89.2% and an F1 score of 90.8%
Fang et al. (17)	DCNN	1,449 patients	Vertebral segmentation and bone mineral density	The minimum average dice coefficients for three testing sets were 0.823, 0.786, and 0.782
Jamaludin et al. (18)	CNN	2,009 patients/12,018 discs	Lumbar MRI radiographic grading	Close to human performance
Yabu et al. (19)	CNN	814 patients/1,624 slices	Osteoporotic Vertebral Fracture (OVF)	AUC 0.949

### Proposing new classification

The level of heterogeneity of clinical feature and treatment options for adult spinal deformity (ASD) is one of the most important features of the condition. There is a lack of an objective classification to guide which patients with ASD may benefit most from surgical treatment and which surgical treatment is likely to yield the best results. In 2019, Ames (20) proposed artificial intelligence-based (AI) hierarchical clustering as a step in a classification scheme to optimize the overall quality, value, and safety of ASD surgery. The study analyzed 570 patients, identified three optimal patient types and four surgical clusters, and the clusters based on patient characteristics and surgical clusters generated 12 subgroups, SRS-22, ODI, SF-36 and the incidence of complications in each subgroup were analyzed 2 years after surgery to enhance preoperative decision-making. In addition, pattern recognition can be treated by education surgeon which patterns can be obtained under the condition of the lowest risk best improve, thereby promoting treatment optimization. In 2021, Durand et al. (21) used an unsupervised self-organizing neural network to classify the overall sagittal spinal and pelvic morphology of adult spinal malformations based solely on sagittal spinal images and independent of pre-measured angles. The study classified 915 adult patients who had preoperative lateral radiographs. The mean spinal shape of six clusters was plotted and found to be correlated with sagittal plane parameters, baseline levels, and operation characteristics. The relationship between sagittal vertical axis (SVA) and proximal junctional kyphosis (PJK) varies with clusters. This study illustrates the value of analyzing the overall spinal shape of all spinal pelvic structures rather than isolated metrics between selected structures. This study represents a major advance in integrating computer vision into a clinically relevant classification system for adult spinal malformations.

### Improving diagnosis rate

#### Based on the ultrasound imaging

Current methods for intraoperative localization and visualization of nerve structures within the psoas muscle are limited and may affect the safety of lateral lumbar interbody fusion (LLIF). The ultrasonic technology enhanced by neural detection algorithm based on artificial intelligence can be used in this work. In 2021, Carson (11) developed image processing and machine learning algorithms using an *in vivo* pig model (50 subjects), and used an ultrasound imaging system to detect the internal and adjacent nerves and other anatomical structures of the lumbar muscle during lateral lumbar surgery. The imaging system's ability to detect and classify anatomical structures was evaluated in subsequent tissue dissection. The mean Dice coefficient score for each tissue type was >80%, the mean specificity of nerve detection was 92%; for bone and muscle, it was >95%. The accuracy of nerve detection was >95%. AI-enhanced ultrasound imaging can provide the important anatomical structures near the visual figure, so as to provide the surgeon with aims to improve the security of key information LLIF surgery.

#### Based on the x-ray

Manual measurement and calculation of a large number of spinal and pelvic parameters on whole spine radiographs in a clinical setting requires considerable time and effort. Therefore, semi-automatic or automatic locating of spinal radiographic anatomical markers and vertebral segmentation on radiographs have been explored for more than ten years. Recently, deep learning has been applied to automatic sagittal imaging parameters measurement, and has good correlation with manual measurement (12, 13, 15, 22, 23). It is worth mentioning that there are ways to better represent spinal alignment because they can evaluate multiple spinal pelvic parameters at once. For example, Galbusera et al. (12) trained 78 different deep learning models to derive 78 anatomical coordinates and six pelvic parameters. Korez et al. (13) could first detect four anatomical structures and then derive five anatomical markers within the detected structures. However, these models may not be able to recognize between similar adjacent anatomical structures, the imaging parameters predicted are not comprehensive enough to cover the entire spinal and pelvic structures, or some of these studies (13, 24–26) often involve segmentation of the image into small pieces and may lose the ability to utilize all relevant anatomical structures of the entire image. For pathological spine images, test data sets are often inadequate in number and diversity, and they may not represent a true clinical picture. Yeh et al. (14) created a dataset of 2,210 radiographs, which is the biggest annotated dataset of all kinds of pathological spine to date. The deep learning model constructed uses the anatomical structure of the entire x-ray film to predict anatomical coordinates and generates various radiological parameters that are well correlated with manual measurements.

In 2018, Wu et al. (15) put forward a new Multi-View Correlation Network (MVC-NET) architecture which can provide a fully automated end-to-end framework for assessing Adolescent Idiopathic Scoliosis (AIS) in multi-view (AP and LAT) x-rays. The results of the experiment on 526 x-ray images from 154 patients indicate an impressive 4.04° Circular Mean Absolute Error (CMAE) in AP Cobb angle and 4.07° CMAE in LAT Cobb angle estimation, which shows the MVC-Net's capability of robust and accurate estimation of Cobb angles in multi-view x-rays. It provides clinicians with an effective, accurate and reliable framework for assessing spinal curvature for comprehensive AIS assessment.

#### Based on the CT

Osteoporosis is characterized by loss of bone mass and damage to bone structure, leading to osteoporosis and deeply increasing the risk of fractures. Fractures caused by osteoporosis are emerging as a primary health issues for the elderly (27) causing severe personal suffering and a social and economic burden. The current clinical standard for assessing fracture risk is Bone Mineral Density (BMD) by Dual x-ray Absorbtiometry (DXA) combined with clinical risk factors. However, less than half of fracture patients are clinically diagnosed with osteoporosis by BMD test (28). In addition to the DXA, other imaging methods have been used to identify fractures in high-risk individuals, including CT-based volumetric BMD and geometry (29, 30) and finite element analysis of CT images (31–33). There are endless researches on CT image analysis and modeling using machine learning method to predict fracture caused by osteoporosis. In 2018, Tomita et al. (16) used a deep convolutional neural network (CNN) to extract radiological features from each slice of CT scan. These extracted features are processed by feature aggregation modules for final diagnosis on full CT scans and detection of OVF at the licensed radiologist level for sporadic CT examinations of the chest, abdomen, and pelvis. Based on the test results of 129 CT scans, the accuracy of the system was 89.2% and the F1 score was 90.8%. This automated detecting system could potentially reduce the time and labor load of radiologists screening for osteoporotic vertebral fractures and reduce the potential for false negative results in the early diagnosis of asymptomatic vertebral fractures. The system can also help improve the diagnosis of osteoporotic vertebral fractures in a clinical setting by pre-screening routine CT examinations and flagging suspicious cases prior to the radiologist's examination. In 2021, Fang et al. (17) applied deep learning to patients with primary osteoporosis and explored an automatic model based on deep convolutional neural network (DCNN), which is used for vertebral segmentation and bone mineral density calculation in CT images. Deep learn-based methods can realize automatic identification of osteoporosis, osteopenia and normal bone mineral density in CT images, which is helpful for clinicians to screen and diagnose opportunistic osteoporosis in CT scans of spine or abdomen.

#### Based on the MRI

In 2017, Jamaludin et al. (18) analysed 12,018 discs in 2,009 patients using a convolutional neural network without a separate segmentation step prior to classification, using disc volume as input and training only on specific disc classification labels to automatically generate lumbar MRI radiographic grading. In this model, the classification of lumbar disc degeneration, disc stenosis, upper/lower marrow changes, spondylolisthesis, and central canal stenosis has achieved close to human performance. On the basis of the work, DeepSPINE Framework took advantage of a great dataset of 22,796 lumbar disc herniated segments to train a convolutional neural network and grade spinal and foraminal stenosis in a multi-task mode in 2018. The studies were more accurate in the classification of lumbar spinal stenosis (84.5%) and lumbar foraminal stenosis (89.0%) than any other published study. Furthermore, the DeepSPINE framework performed equally to human evaluators in detecting and grading lumbar spinal stenosis and foraminal stenosis. In 2021, Yabu et al. (19) constructed nine neural network models to detect fresh osteoporotic vertebral fractures and constructed an optimal model using an integrated approach. Tools for automated detection of osteoporotic cone fractures have previously been reported (16). However, these tools only assess the existence of osteoporotic vertebral fractures on CT images, not the freshness of the fractures In this study, 1,624 T1-weighted MRI images from 814 patients with fresh osteoporotic vertebral fractures were used to train and verify the model. Finally, the area under ROC curve (AUC) was 0.949. The diagnostic accuracy, specificity and sensitivity of the model were comparable to those of two independent doctors.

In most instances artificial intelligence cannot directly apply the text image generated by radiology department. Medical reports are usually unstructured text in natural language, but they are difficult to access and are not suitable for annotation in artificial intelligence model training and testing. Most studies using deep learning techniques to identify spinal parameters have been recorded, but their accuracy and consistency are limited compared with human behavior. At the same time, the graphics technology related to spinal image is mainly committed to developing segmentation methods, and the results will be greatly affected by inevitable noise. Automatic measurement of spinal parameters is the application of artificial intelligence in medicine and orthopedics, and is considered to be an significant tendency in the next few years. Breakthroughs in machine vision will contribute to the development of medical imaging. Improving diagnostic criteria for spinal diseases through “human-machine” integration will help improve medical standards and reduce medical costs.

## Surgical treatment

Artificial intelligence, especially deep learning, is promoting the development of several fields. Virtual reality (VR) and augmented reality (AR) are being expected to benefit more from advances in AI. The advantages of deep learning in object tracking and segmentation and video resolution enhancement can reduce the computing power required by AR and VR systems, reduce the cost of hardware and software, improve equipment performance and enable new functions. These abilities have significantly increased the extent of applications of AI and AR in spinal surgery, which lead to the approval of the first AR assisted spinal surgery systems by the US Food and Drug Administration in June 2020.

In spinal surgery, augmented reality AI systems have been used in pedicle screw placement (34, 35). Proper placement of pedicle screws is critical to the strength and durability of the screws. In 2017, Ma et al. (36) from Tsinghua University proposed a original AR based navigation system for pedicle screw placement. They take advantage of ultrasound to connect 3D anatomical markers to CT images, using Kirschner wire (K-wire) instead of pedicle screws, and compared their system with a skin marker tracer system. Their ultrasound AR system showed an average positioning error of 3.79 mm and an average Angle error of 4.51°, while the skin marker tracer system showed an average positioning error of 5.18 mm and an Angle error of 5.89°.

Although AR and VR still have some challenges. The bond of robot-assisted navigation systems with AR and AI can produce quick and accurate navigation systems. AI, wearable devices and AR can be integrated to provide real-time feedback to surgeons at the time of surgery. Combining AI, AR, and VR can facilitate remote instruction and its integration with wearables, while AI and surgical robots enable remote and semi-automatic surgery. Through collaboration between clinicians and engineers, we will have the ability to bring all of these fields together over the next decade to profoundly improve the way spine surgery is performed.

## Predict the prognosis

Machine learning as an emerging technology, its advantage in medical diagnosis and imaging has been well proven (37). However, it has recently been applied to epidemiological data sets to predict a variety of health-related outcome (Table 3). Just like simple regression models, machine learning algorithms can predict outputs given some inputs (43). However, statistical knowledge used in machine learning are more complex in generating predictions from input data. Machine learning has a lot of advantages including the capacity to process big data sets and capture nonlinear relationships compared with traditional statistical models (43). And its superiority over the traditional model has been proved in the literature (44).

**Table 3:** AI and ML in the task of predicting the prognosis.

Author	Models	Dataset	Type of outcome	Result
Khan et al. (38)	SVM	757 patients	Change in mJOA at 1 year	AUC 0.834
Karhade et al. (39)	Bayes	1,790 patients	30-day mortality	AUC 0.782
Kuris et al. (40)	NN	63,533 patients	30-day readmission	AUC 0.64–0.65
Karhade et al. (41)	Stochastic Gradient	2,737 patients	Sustained postoperative opioid prescription	AUC 0.81
Wang et al. (42)	ANN	12,492 patients	Complications	AUC 0.748

In 2021, Khan et al. (38) developed a machine learning model to predict the deterioration of functional status of patients with cervical spondylotic myelopathy after surgical intervention, and identified important predictors of imaging prognosis as a potential tool for guiding surgical decision making. 757 patients were enrolled in the study. After using 8:2 train-test segmentation to the data set, they trained, optimized and tested many ML algorithms to assess algorithm performance and identify predictors of worse mJOA after 1 year. The highest-performing ML algorithm was a polynomial support vector machine which showed good calibration and discrimination on the testing data, with an area under the receiver operating characteristic curve of 0.834 (accuracy: 74.3%, sensitivity: 88.2%, specificity: 72.4%). Vital predictors of functional decline at 1 year included initial mJOA, male sex, duration of myelopathy, and the presence of comorbidities. The development of these algorithms provides a reference for clinicians to identify and timely manage patients at risk for further neurological deterioration after surgery. In 2018, Karhade et al. (39) assessed the efficacy of several machine learning models in predicting 30-day mortality after spinal metastasis surgery. The algorithm has the best performance in recognition, calibration and overall performance, and is integrated into an open access web application. As the volume of oncology data continues to grow, establishing learning systems and deploying them as accessible tools may greatly strengthen prediction and management. Kevin et al. (45) used a Bayesian classification algorithm to predict 30-day mortality after spinal tumor resection from the National Surgical Quality Initiative Program. The algorithm exceeds the predictive power of the National Surgical Quality Initiative mortality probability Calculator. Multivariate regression analysis showed that smoking history, chronic obstructive pulmonary disease, cancer cell spread, history of hemorrhagic disease, dyspnea, and low albumin levels were strongly associated with 30-day mortality. As the model continues to learn from input patient data, its accuracy increases. Patient outcomes can be improved by using the algorithm to identify high-risk individuals early and applying this data to preoperative decision-making as well as patient selection and education. In 2020, Nida et al. (46) from Harvard Medical School, used the American College of Surgeons National Surgical Quality Improvement Program database to develop and validate preoperative predictive variables for patients with adverse events occurring within 30 days after degenerative lumbar spondylolisthesis surgery. The predictive probabilities obtained from the best predictive models were uploaded to a publicly accessible website. It is proved that it is feasible to develop machine learning algorithms from large data sets for patient consultation and surgical risk assessment.

Readmission within 30 days of surgery can impose a heavy financial burden on clinicians and hospitals, and sometimes lead to negative outcomes for patients. Previous studies have identified risk factors for readmission, but conclusions about specific patients remain vague. Kuris et al. (40) used the American College of Surgeons National Surgical Quality Improvement Program database to developed a neural network model to predict 30-day readmission for 63,533 patients who underwent anterior, lateral, or posterior lumbar fusion surgery with area under the curve values of 0.64–0.65. Multivariate regression showed that age >65 years and American Society of Anesthesiologists(ASA) class > II were associated with increased risk for readmission for all three procedures. A study that also used the database identified that advanced age (50 years), anterior and posterior spinal fusion surgery, elevated American Society of Anesthesiologists grade, and isolated tumor diagnosis are risk factors for readmission (47). A separate analysis from national and single-institution registries showed that higher-than-average and upper quartile surgery duration and Medicare/Medicaid insurance were also related to an increased risk of readmission.

In addition to predicting functional improvements and complications after surgery, the new AI technology can also make recommendations for the medications after surgery. In 2019, Karhade et al. (41) proposed solutions for opioid abuse, particularly the continued use of opioids after spinal surgery. Although many demographic and clinical features have previously been identified as prognostic factors for continued opioid use after spinal surgery, there is currently no predictive algorithm for preoperative risk stratification of patients. The model can stratify the risk of these patients before surgery, making it possible to intervene early to reduce the likelihood of long-term opioid use.

In recent years, ACDF has become popular in ambulatory surgical Settings. There is currently no agreed risk stratification tool to identify patients who might be safe candidates for ambulatory ACDF. In 2021, Wang et al. (42) used an artificial neural network model to stratify the risk of ACDF in 12,492 patients from the National Surgical Quality Improvement Program database. Patients would be regarded as “unsafe” for outpatient surgery if they suffered any complication within a week of the index operation. The ANN showed an AUC of 0.740, which was significantly higher than the AUCs of ASA (*P* < 0.05). Advanced age, low hemoglobin, high international normalized ratio, low albumin, and poor functional status were considered to be significant in the multivariable predictive model.

Clinicians can provide personalized treatment and counseling methods for patients by accurately predicting outcomes based on a patient's phenotype and clinical presentation. The integrated prediction in the field of degenerative disease of the spine will improve the decision of prognosis and the subsequent delivery of personalized medicine based on those prognosis. Considering the potential consequences of overestimating or underestimating the results of such studies for clinical decision making, the improper application of machine learning is a major bioethical challenge. Solutions to this problem include receiving the machine learning black box and testing. In short, we need a healthy skepticism of machine learning and a willingness to appreciate its methodology.

## Decision support systems

Decision support systems, a widely used predictive analytics application in clinical practice, utilize the predictive power of models to support clinical decision making by providing personalized predictions. In 2018, Varghese et al. (48) aimed to build a learning-based predictive model to understand the sensitivity of pedicle-screw holding power to various factors. Of the various machine-learning techniques, the random forest regression model performed well in predicting the pullout strength with a correlation coefficient of 0.99 between the observed and predicted values. The model was able to predict the holding power of a pedicle screw for any combination of density, insertion depth, and insertion angle for the chosen range. Similarly, in 2019, Khatri et al. (49) used an experimental dataset of 48 data points as training data to construct a model based on different machine learning algorithms. They also used the L9 orthogonal array of Taguchi Design of Experiments to obtain the best combination of parameters for predicting the pullout strength. Finally, random forest performed the best with a correlation coefficient of 0.96. The model developed in this study can help surgeons be better prepared for surgery and the decision would be based on objective, rather than subjective parameters.

## Future prospectives

The applications of AI technologies in healthcare, especially regarding tools with a direct clinical impact such as those aimed at clinical decisions support systems, should be in a better monitoring environment for ethical reasons. The use of AI in healthcare also raises serious concerns about data privacy and security due to the massive amount of clinical and imaging data required for training and validation of the tools. In the future, we should pay more attention to data security to ease people's concerns about privacy disclosure.

## Summary

At present, the application of artificial intelligence in spine surgery is still in its infancy stage, facing many challenges, such as scattered data, integration degree is not enough, and clinical conversion efficiency is low. Better integration, mining and management of unstructured data will contribute to the further development of artificial intelligence in spine surgery. In the future, the application of artificial intelligence and machine learning technology in spinal surgery will be conducive to improving the level of medical diagnosis and treatment, optimizing the medical process, developing the clinically assisted decision-making system, and alleviating the pain of patients and reducing the social and economic burden.
